# Scaling crossover in thin-film drag dynamics of fluid drops in the Hele-Shaw cell

**DOI:** 10.1038/srep31395

**Published:** 2016-08-26

**Authors:** Misato Yahashi, Natsuki Kimoto, Ko Okumura

**Affiliations:** 1 Department of Physics and Soft Matter Center, Ochanomizu University, 2–1–1, Otsuka, Bunkyo-ku, Tokyo 112-8610, Japan

## Abstract

We study both experimentally and theoretically the descending motion due to gravity of a fluid drop surrounded by another immiscible fluid in a confined space between two parallel plates, i.e., in the Hele-Shaw cell. As a result, we show a new scaling regime of a nonlinear drag friction in viscous liquid that replaces the well-known Stokes’ drag friction through a clear collapse of experimental data thanks to the scaling law. In the novel regime, the dissipation in the liquid thin film formed between the drop and cell walls governs the dynamics. The crossover of this scaling regime to another scaling regime in which the dissipation inside the droplet is dominant is clearly demonstrated and a phase diagram separating these scaling regimes is presented.

Dynamics of liquid drops is familiar in daily life: we observe rain drops rolling on a new umbrella, honey dripping off from a spoon, and oil droplets floating on the surface of vegetable soup and so on. Such everyday phenomena are in fact important not only in physical sciences^1^,^2^,^3^,^4^,^5^,^6^,^7^ but also in a variety of practical issues such as ink-jet printing^8^, microfluidics manipulations^9^,^10^, and emulsification, formation of spray and foams^11^,^12^,^13^. From such phenomena familiar to everybody, researchers have successfully extracted a number of scaling laws representing the essential physics^14^, which include scaling laws associated with the lifetime of a bubble in viscous liquid^15^,^16^ and contact dynamics of a drop to another drop^17^,^18^ or to a solid plate^19^,^20^. Here, we report on a crossover of two scaling regimes experimentally revealed for viscous friction acting on a fluid drop in a confined space. In particular, we study the descending motion (due to gravity) of an oil droplet surrounded by another immiscible oil in a Hele-Shaw cell. The friction law thus revealed is nonlinear and replaces the well-known Stokes’ law in the Hele-Shaw cell geometry.

A closely related topic of the rising bubble in a Hele-Shaw cell is theoretically discussed by Taylor and Saffman in a pioneering paper^21^ in 1958 (earlier than the Bretherton’s paper on bubbles in tubes^22^,^23^). The solution of Taylor and Saffman was further discussed by Tanveer^24^. There are many other theoretical works on fluid drops in the Hele-Shaw cell geometry, notably in the context of the topological transition associated with droplet breakup^25^,^26^,^27^,^28^. As for experimental studies, a number of researchers have investigated the rising motion of a bubble in a Hele-Shaw cell^29^,^30^,^31^. However, unlike the present study, systematic and quantitative studies in a constant velocity regime have mostly concerned with the case in which there is a forced flow in the outer fluid phase and most of the studies have been performed with the cell strongly inclined nearly to a horizontal position (one of a few examples of the case with the cell set in the upright position but with external flow^32^ demonstrates relevance of the present work to important problems in petroleum industry, such as the suction of crude oil from the well).

One of the features of the present study compared with most of previous ones on the dynamics of fluid drops in a Hele-Shaw cell is that in the present case the existence of a thin liquid film surrounding a fluid drop plays a crucial role: In many previous works, the existence of such thin films is not considered. In this respect, the present problem is closely related to the dynamics governed by thin film dissipation such as the imbibition of textured surfaces^33^,^34^,^35^,^36^,^37^,^38^. In this sense, our problem is quasi two-dimensional, although the geometry of the Hele-Shaw cell is often associated with a purely two-dimensional problem.

## Experiment

**Figure 1:**
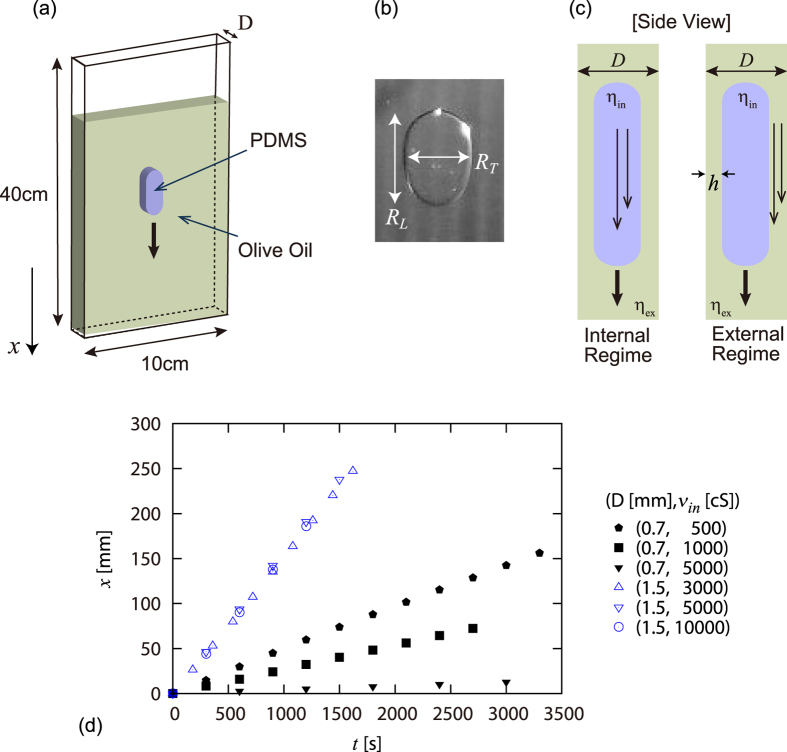
(**a**) Experimental setup. Gravity is acting in the *x*-direction. (**b**) Front view of a PDMS drop of kinematic viscosity *ν*_*in*_ = 1000 cS going down in olive oil in a Hele-Shaw cell of thickness *D* = 2 mm. (**c**) Magnified side views of droplets with the velocity gradient in the internal end external regimes. (**d**) Position of the PDMS drop *x* as a function of elapsed time *t*.

We fabricated a Hele-Shaw cell of thickness *D*^16^,^18^,^39^ and filled the cell with olive oil (150-00276, Wako; kinematic viscosity *ν*_*ex*_ = 60 cS and density *ρ*_*ex*_ = 910 kg/m^3^). This oil plays a role of an external surrounding liquid for a drop of poly(dimethylsiloxane) (PDMS) to be inserted at the top of the cell using a syringe (SS-01T, Termo). We observe the inserted drop going down in the cell, as illustrated in Fig. 1(a), because of the density difference Δ*ρ* = *ρ*_*in*_ − *ρ*_*ex*_ > 0. The drop density *ρ*_*in*_ depends on its kinematic viscosity *ν*_*in*_ only slightly (see the details for Methods). The drop size is characterized by the cell thickness *D* and the width *R*_*T*_, i.e., the size in the direction transverse to that of gravity (see Fig. 1(b)), which is slightly smaller than the size in the longitudinal direction, *R*_*L*_. As shown in Fig. 1(c), a thin film of olive oil exists between a cell plate and the surface of the drop. We can think of two limiting cases for the distribution of liquid flow: (1) Internal Regime: The velocity gradient is predominantly created in the internal side of the droplet as in the left illustration. (2) External Regime: The gradient is predominantly exists in the external side of the droplet as in the right.

The width and height of the cell are 10 cm and 40 cm, respectively, and are much larger than the drop size to remove any finite size effects in the direction of width and height. The cell is made of acrylic plates of thickness 5 mm, to avoid thinning deformation of the cell due to the effect of capillary adhesion^14^.

We took snapshots of the descending drop at a regular time interval using a digital camera (Lumix DMC-G3, Panasonic) and a camera controller (PS1, Etsumi). The obtained data were analyzed with the software, Image J, to obtain the position as a function of time to determine the descending velocity of the drop. Some examples are shown in Fig. 1(d). This plot show the following facts. (1) The descending motion can be characterized by a well-defined constant velocity (to guarantee a long stationary regime, the cell height is made significantly larger (40 cm) than the drop size; because of a small density difference, the constant-velocity regime starts after a long transient regime). (2) The descending velocity is dependent on the kinematic viscosity of the internal liquid of the drop *ν*_*in*_ for the thinner cell (*D* = 0.7 mm) as predicted in the previous study^40^, which is not the case for the thicker cell (*D* = 1.5 mm); These examples clearly demonstrate the existence of a novel scaling regime different from the one discussed in the previous study^40^.

In the present study, the dependence of the descending velocity on the drop size is negligible. In the previous study^40^, it was found that the descending speed of drops is dependent on *R*_*T*_ for *R*_*T*_/*D* < 10 if a glycerol drop goes down in PDMS oil. However, in the present combination (i.e., a PDMS drop going down in olive oil), we do not observe a significant dependence on *R*_*T*_ in our data even for fairly small drops, whereas *R*_*T*_ is in the range 1.31 < *R*_*T*_/*D* < 15.8 in the present study (the size dependence in the previous study may be caused by the polarity of the glycerol aqueous solution: We expect that if the liquid is polar, the drop may subject to electrostatic (attractive) force from the acrylic cell plates and this effect tends to make the drop less mobile). The data analysis below neglects any possible small dependences of the velocity on the drop size.

## Theory

At the level of scaling laws, the characteristic energy scales are given as follows. The gravitational energy gain for the descending drop per unit time is expressed as


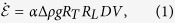


where *α* is a numerical coefficient. The viscous dissipation per unit time in the internal regime discussed above (Fig. 1(c) left) is written as


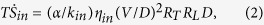


where *α*/*k*_*in*_ is a numerical coefficient. Strictly speaking, because of the existence of the thin film of thickness *h* (Fig. 1(c)), the velocity gradient *V*/*D* in the above expression should be replaced with *V*/(*D* − 2*h*), which is not essential, however, because the relation *D* ≫ *h* is well satisfied in the present study (see the next section). The viscous dissipation per unit time in the external regime discussed above (Fig. 1(c) right) is given as


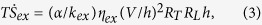


where *α*/*k*_*ex*_ is a numerical coefficient.

In the internal regime the velocity is given by the balance between 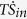 and 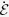,


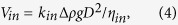


whereas in the external regime the velocity is given by the balance between 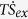 and 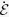,


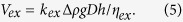


The thickness of the thin film formed between the drop and cell plates may be given by the law of Landau, Levich and Derjaguin (LLD),


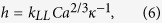


where the numerical coefficient is of the order of unity^14^ (*k*_*LL*_ = 0.94, in the original papers^41^,^42^). Here the capillary length *κ*^−1^ is defined as 
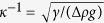
, which is smaller than the cell thickness *D* (If otherwise, the length *κ*^−1^ is replaced with *D* with the coefficient *k*_*LL*_ = 1.337^43^). The capillary number *Ca* is defined as


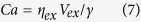


Removing *h* from Eqs (5) and (6), we obtain another expression for the velocity in the external regime,


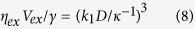


with *k*_1_ = *k*_*ex*_*k*_*LL*_. Removing *V*_*ex*_ from Eqs (5) and (6), we obtain an expression for the thickness of the thin film,


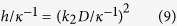


with 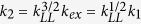.

The condition for the internal regime is given by 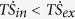, which leads to the equation, *η*_*ex*_/*η*_*in*_ > *k*_3_*D*/*κ*^−1^. In other words, the phase boundary between the internal and external regimes is given by


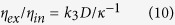


with 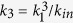. This means that the phase boundary between the internal and external regime is a straight line with the slope *k*_3_ on the plot of *η*_*ex*_/*η*_*in*_ as a function of *D*/*κ*^−1^.

## Experiment and Theory

**Figure 2:**
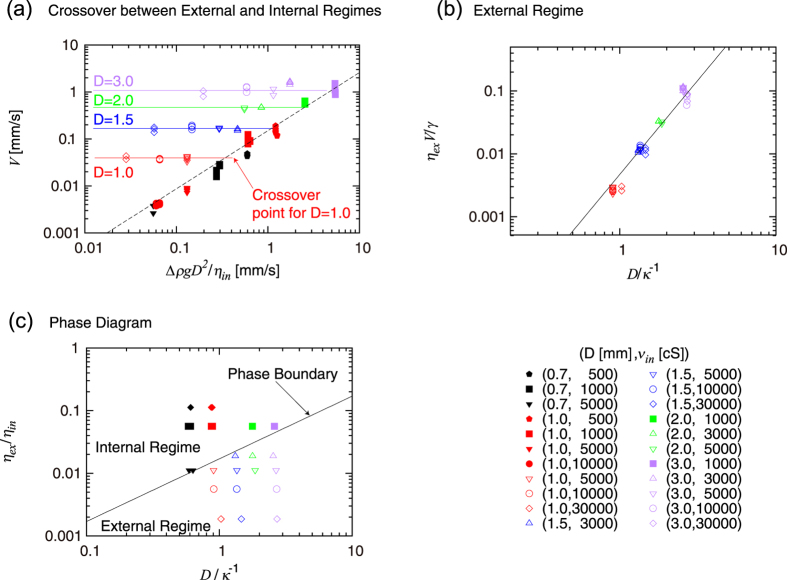
(**a**) Plot of *V* vs. Δ*ρgD*^2^/*η*_*i*_. The data in the internal regime are on the dashed line, whereas the data in the external regime are on horizontal lines for each cell thickness *D*. The crossover between the two regimes would occur at the cross point of the dashed line and each horizontal line. (**b**) Plot of *η*_*ex*_*V*/*γ* vs *D*/*κ*^−1^, confirming the external regime. (**c**) Plot of *η*_*ex*_/*η*_*in*_ vs *D*/*κ*^−1^, showing the phase diagram for the two scaling regimes. Throughout (**a–c**), the data in the external and internal regimes are represented by open and filled symbols, respectively. The color and shape of the symbols are fixed for a given *D* and a given *ν*_*in*_, respectively.

The experimental data for the descending velocity of drops *V* are plotted as a function of Δ*ρgD*^2^/*η*_*in*_ in Fig. 2(a). In view of Eq. (4), the data points in the internal regime would be on a straight line of slope 1. This is almost true: there is a series of data well on the dashed line of slope close to one. Naturally, there is a slight deviation from the theory: the slope of the straight dashed line obtained by a numerical fitting is in fact 1.24 ± 0.06, a value slightly larger than one, but the coefficient corresponding *k*_*in*_ is 0.150 ± 0.015, the order of magnitude of which is consistent with the scaling arguments.

Some detailed remarks for the above arguments are as follows. (1) Even in the previous study^40^ in which the internal scaling regime was confirmed for the first time, the scaling regime described by Eq. (4) was shown with some deviations, similarly to the present case (whereas another scaling regime first established in the previous paper^40^ is almost perfectly demonstrated). (2) We note here that the data represented by the red filled circle and red filled inverse triangle are exceptional ones and their seemingly strange behavior will be explained in Discussion. (3) We have confirmed that even if we replace *D* with *D* − 2*h* in the analysis (by using the thickness *h* estimated from Equation (6)) when *D* is used as a length scale characterizing the viscous gradient (*i.e*., when D is used in the expression *V/D* in Eq. (2)), any visible differences are not introduced into the plots given in Fig. 2 (This correction could be motivated by considering the existence of thin films surrounding the drops as in Fig. 1(c) as mentioned above).

In Fig. 2(b), it is shown that some of the data we obtained clearly satisfy Eq. (8), which describes the external regime. In Fig. 2(b), we collected the data points that are off the dashed line of slope close to one in Fig. 2(a) and that are thus ruled out from the internal regime. The data thus selected and plotted in Fig. 2(b) are almost on the straight line of slope 3 in accordance with Eq. (8). The straight line is obtained by a numerical fitting with fixing the slope to 3.0; as a result of this fitting, the coefficient is given as *k*_1_ = 0.167 ± 0.003, the order of magnitude of which is consistent with the scaling arguments.

We confirm this scaling law in Eq. (8) also in Fig. 2(a). In the light of Eq. (8), the data in the external regime for a given *D* should take almost the same values, because *η*_*ex*_ and *γ* are both constant and *κ*^−1^ is almost constant (note that Δ*ρ* is almost constant) in the present study. In fact, in Fig. 2(a), the data points for a fixed *D* that are off the dashed line, which data are shown to be in the external regime in Fig. 2(b), take almost a constant value, that is, they are located almost on a horizontal line. This fact also confirms that the data in question are independent of *η*_*in*_, that is, they are certainly not in the internal regime. Strictly speaking, the data labeled as a given *D* can have slightly different measured values of *D* (see Methods), which is the main reason the data for a “given” *D* that are off the dashed line in Fig. 2(a) slightly deviate from the straight horizontal line corresponding the *D* value.

The scaling law in Eq. (8) can be confirmed in Fig. 2(a) in a still another way. The open marks of the same shape, say diamond, but with different colors (that are the data for a given *ν*_*in*_ but with different *D*) are almost on a straight line of a slope close to one (This slope may seem to be slightly larger than one, which may be because of the uncertainty on the cell spacing *D* as already mentioned in the last sentence of the paragraph just above this one, or because the exponent 3 in Equation (8) may be in fact slightly larger than 3 in a more complete theory beyond the present arguments at the level of scaling laws). For a such series of data, the velocity *V* scales with *D*^3^ according to Eq. (8), thus when plotted as a function of *D*^2^ as in Fig. 2(a), the quantity linearly scales with *D*, as reasonably well confirmed.

The phase diagram based on Eq. (10) is shown in Fig. 2(c), in which we plot all the data (except for the special data mentioned above), to demonstrate further consistency of the present arguments. As expected from Eq. (10), we can indeed draw a straight line of slope 1 on Fig. 2(c), which divides the internal and external regimes; Above the straight line of slope 1 in Fig. 2(c) lie the data in the internal regime described by Eq. (4), i.e., the data on the straight dashed line in Fig. 2(a); Below the straight line in Fig. 2(c) lie the data in the external regime described by Eq. (8), i.e., the data on the straight line in Fig. 2(b). The coefficient *k*_3_ of Eq. (10), i.e., the line dividing two regimes shown in Fig. 2(c), is *k*_3_ = 0.017, the order of magnitude of which is consistent with the scaling arguments in a profound sense: The numerical coefficient, *k*_*in*_, *k*_1_, and *k*_3_, are predicted to satisfy the relation 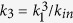, and this relation is satisfied at a quantitative level in the present analysis (0.017 vs 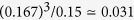). This quantitative agreement is indeed quite satisfactory, if we consider slight deviations of the data from the predicted theory. For example, the value 0.15 used in the estimation in the parentheses is not the value of *k*_*in*_ itself (the precise definition of *k*_*in*_ is the coefficient appearing in Eq. (4), *V*_*in*_ = *k*_*in*_Δ*ρgD*^2^/*η*_*in*_, but the value of *k*_*in*_, 0.15, used in the above is in fact the value of the coefficient 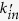 appearing in the relation 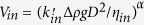 obtained when the data corresponding to the internal regime in Fig. 2(a) are numerically fitted by this relation with *α* determined to be not equal to one but close to 1.24, as mentioned in the first paragraph in Experiment and Theory). In addition, the exponent in (8) might also be slightly deviated from 3 as suggested in the paragraph just above this one.

The crossover from the internal to external regime can explicitly be seen in the data for *D* = 1.0 mm (red data) in Fig. 2(a). As *η*_*in*_ decreases from the left-most data for *ν*_*in*_ = 30000 cS (red open diamonds) to the data for *ν*_*in*_ = 5000 cS (red open inverse triangle), the velocity is independent of *ν*_*in*_, which reveals that the three data on the horizontal line are in the external regime. However, the data for *ν*_*in*_ = 1000 cS and *ν*_*in*_ = 500 cS are on the straight dashed line with a slope close to one, which confirms that these two data are in the internal regime. Since the phase boundary expressed by Eq. (10) is obtained also by equating *V*_*in*_ and *V*_*ex*_ in Eqs. (4) and (8), the crossover between the two regimes occurs in Fig. 2(a) near at the cross point between the horizontal line connecting the data on the external regime for a given *D* and the straight dashed line of a slope close to one representing the internal regime.

The behavior of the data close to the crossover points are quite intriguing. The data for *D* = 2.0 mm and 3.0 mm at *ν*_*in*_ = 1000 cS (green filled square and purple filled square) are located at the position close to the phase boundary in Fig. 2(c) (and the data have already been confirmed to be in the internal regime in Fig. 2(a): in this plot, these data points are reasonably well on the dashed line). We have confirmed that, when these two data are plotted in Fig. 2(b), they are nearly on the straight line of slope close to 3 in Fig. 2(b). The two points can be described by both Eqs (4) and (8), which is reasonable because they are nearly on the phase boundary. However, this is not always the case. The data for *D* = 0.7 mm and *ν*_*in*_ = 5000 cS (black filled inverse triangle) and for *D* = 1.5 mm and *ν*_*in*_ = 3000 cS (blue open triangle) are also positioned close to the phase boundary in Fig. 2(c). However, the former is rather in the internal regime and the latter rather in the external regime. This is in a sense logical because the blue open triangle is rather away from the crossover point for *D* = 1.5 mm in Fig. 2(a) but this is not the case for black filled inverse triangle. In general, how quickly the crossover occurs seems to be a subtle problem.

## Discussion

**Figure 3:**
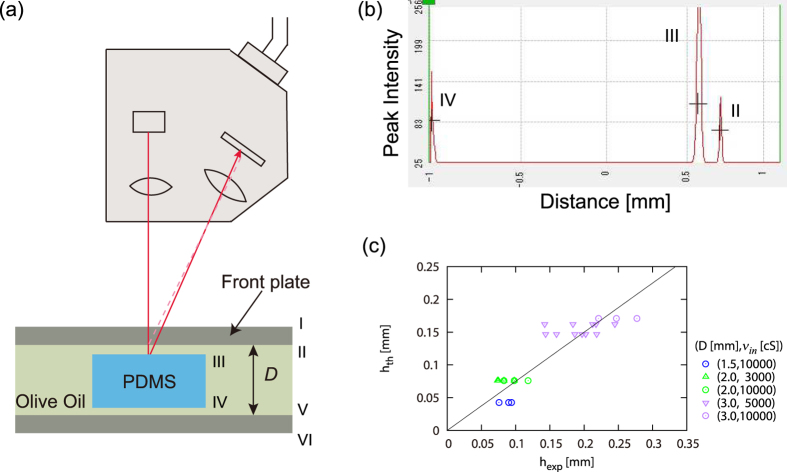
(**a**) Setup for the thickness measurement. (**b**) Example of the screen shot of the three peaks (the left to the right) originating from interface IV, III and II (see the text for the details). The distance between IV and III is obtained by multiplying *n*_*PDMS*_ to the distance, whereas that between III and II is obtained via *n*_*olive*_ instead. (**c**) Plot of the experimentally obtained value of the thin film thickness *h*_*exp*_ vs. the theoretical estimation *h*_*th*_. The theoretical value *h*_*th*_ is obtained by Eq. (9) with the coefficient 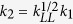 with *k*_1_ = 0.15 and *k*_*LL*_ = 0.94.

The direct measurement of the thickness *h* supports the above analysis. We used a laser distance sensor (ZS-HLDS2 + ZS-HLDC11 + Smart Monitor Zero Pro., Omron), as illustrated in Fig. 3(a). The measurement is extremely delicate and difficult, because we have six reflective planes I to VI with significantly different strengths of reflection where the target two reflections II and III are the smallest and the second smallest among them (see Fig. 3(b)). The six surfaces are the front and back surfaces of the front cell plate (interface I and II), the front and back interfaces between olive oil and the PDMS drop (interface III and IV), and the front and back surfaces of the back cell plate (interface V and VI). To determine *h*, we need to detect reflection from interface II and III, where the reflection from II is small compared with that of III (see Fig. 3(b)) and significantly small compared with that of I, because the refractive index of olive oil is *n*_*olive*_ = 1.47, that of acrylic plate is *n*_*acr*_ = 1.491, that of PDMS oil is *n*_*PDMS*_ = 1.403 and that of air is *n*_*air*_ = 1. Furthermore, the object (the descending drop) is moving. In spite of these experimental difficulties, we obtained a reasonably good correlation between the measured thickness and the experimentally obtained value as shown in Fig. 3(c), by virtue of various efforts (for example, in the screen shot Fig. 3(b), the two target peaks are intensionally positioned off-center because the precision of measurement becomes the maximum when the reflection angle is the largest). Here, the slope of the line obtained by a numerical fitting is 0.749 ± 0.027 (the slope here is not the exponent but the coefficient for the linear relationship), the order of magnitude of which is consistent with the scaling argument.

Exceptional data mentioned above reveal an intriguing phenomenon. In Fig. 2(a), the data for *D* = 1 mm and for *ν*_*in*_ = 10000 cS are represented by two different marks, the red filled circle and the red open circle, with the former described by the internal regime and the latter by the external regime. The data for *D* = 1 mm and for *ν*_*in*_ = 5000 cS are also categorized into two filled and open symbols. The experimental difference in acquiring these two different types (filled and open symbols) of data obtained for identical drop viscosity and cell spacing is that, when the drop goes down on the same path multiple times in the same cell, the first drop is in the external regime (open marks) whereas the drop going down after the first one is always in the internal regime (filled marks). This apparently mysterious effect is quite reproducible and is understood by considering a possibility of mixing of olive oil and PDMS at the surface of the drops. For the first drop, such a mixing effect is negligible and the drop is governed by the dynamics of the external regime. However, after the first one, because of the mixing effect, the viscosity of the thin film surrounding the drop increases (because *ν*_*in*_ ≫ *ν*_*ex*_) so that making a velocity gradient in the external thin film is no longer favored in terms of energy and instead the velocity gradient inside the drop is favored to realize the dynamics in the internal regime. Because of this reason, the red filled circles and the red filled inverse triangles are not shown in the phase diagram given in Fig. 2(c). This seemingly mysterious behavior tends to be suppressed if the viscosity is too small (because the “external” viscosity does not get sufficiently viscous), or too large (because the mixing is not sufficiently effective). This is why we observed this phenomenon only for the two values of viscosity.

The present study suggests that Stokes’ drag friction *F* = 6*πη*_*ex*_*VR* for a solid sphere of radius *R* surrounded by a viscous liquid of viscosity *η*_*ex*_ is replaced in the external regime of the Hele-Shaw cell geometry by


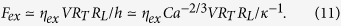


This expression possesses a nonlinear dependence on the velocity *V* due to the extra *V* dependence contained in the capillary number *Ca*. This is strikingly different from the two other expressions for the drag force: 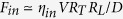 and 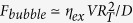, which are both linear in velocity; The former corresponds to the internal regime in the present study, whereas the latter corresponds to the case in which the dominant dissipation is the one associated with the velocity gradient *V*/*D* in the surrounding external liquid^40^. The viscous friction forces including the nonlinear friction in Eq. (11) are relevant to the dynamics of emulsion, foam, antifoam and soft gels^13^,^44^,^45^, in particular, nonlinear rheology of such systems^46^,^47^,^48^.

We intentionally used several times the phrase, “ the order of magnitude of which is consistent with the scaling argument,” which may be vague compared with an expression like, “ being of order one further supports the scaling argument.” The reason we used the seemingly vague expression is that whether a coefficient for a scaling law is of the order of one or not is in fact a subtle issue. Depending on the problem or on the definition of the coefficient, the orders of magnitude can be fairly larger or smaller than one. An example of such a case can be given by exploiting the relation, 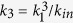, given above: The three coefficients, *k*_1_, *k*_3_, and *k*_*in*_ are all coefficients for some scaling laws so that, for example, *k*_1_ and *k*_*in*_ can be 5 and 1, respectively, but this example implies *k*_3_ is much larger than one (*k*_3_ = 5^3^).

In the present study, the consistency of the whole scaling arguments is checked in several ways, which clearly deepens our physical understanding. For example, a new scaling regime is demonstrated through a clear data collapse (Fig. 2(b)), and the crossover of this regime to another is shown (Fig. 2(a)), which is completed by the phase diagram (Fig. 2(c)) and a separate measurements on thin-film thickness (Fig. 3(c)). In addition, data arrangements in the crossover diagram (Fig. 2(a)) are interpreted from various viewpoints, confirming the consistency of the arguments.

## Conclusion

In summary, we show in Fig. 2(b) the existence of a novel scaling regime for the descending velocity of a drop surrounded by thin external fluid in the Hele-Shaw cell, in which regime the viscous dissipation in the thin film is essential. This regime corresponds to a nonlinear form of viscous drag friction. In this regime, the thickness of the film is determined by the law of LLD, as directly confirmed in Fig. 3(c). The crossover between this regime and another regime in which the viscous dissipation in the internal side of the drop governs the dynamics is shown in Fig. 2(a). The phase boundary between the two regimes are given in Fig. 2(c).

There are some other scaling regimes for the viscous drag friction in the Hele-Shaw cell geometry with the existence of thin films surrounding a fluid drop. For example, the dissipation associated with the velocity gradient *V*/*D* in the internal drop liquid has been revealed to be important for a rising bubble in the Hele-Shaw cell^40^. The dissipation associated with the dynamic meniscus (in the context of LLD theory^14^,^41^,^42^) formed in the external thin-film has been found to be important in a non Hele-Shaw cell geometry^49^. In addition, the present external regime will give another scaling law if the capillary length *κ*^−1^ is, unlike in the present study, larger than the cell thickness *D*.

Confirmation of such other regimes for viscous drag friction in the Hele-Shaw cell geometry, as well as crossovers among various scaling regimes would be explored in future studies. The simple friction laws for confined fluid drops and the crossover between them revealed in the present study (and in future studies) are relevant to fundamental issues including rheology of foam and emulsion, as well as applications such as in microfluidics.

## Methods

The density of PDMS oil *ρ*_*in*_ slightly depends on viscosity: (1) 970 kg/m^3^ for the kinematic viscosities *ν*_*in*_ = 500, 1000, and 3000 cS (SN-4, SN-5, and SN-6, As One). (2) 975 kg/m^3^ for *ν*_*in*_ = 5000 and 10000 cS (SN-7 and SN-8, As One). (3) 976 kg/m^3^ for *ν*_*in*_ = 30000 cS (KF-96H, ShinEtsu).

The cell thickness *D* is controlled by spaces, and is directly measured using the laser distance sensor (ZS-HLDS5, Omron) for most of the cells. In all the figures of the present study, for simplicity, the cell thickness *D* is represented by an approximate value, which is slightly different from measured values. For some of the data the measurement of *D* was not performed and in such a case an approximate value of *D* is used, instead of measured values, to plot the data points, which does not cause serious difficulties in analyzing and interpreting the data. This is because the difference between the *D* value used for labeling and the measured value of the cell thickness is rather small.

The interfacial tension between PDMS and olive oil was measured by using pendant drop tensiometry. It is recently discussed that measured values for pendant drops are dependent on Bond number and Worthington number, with both scaling with 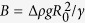 (*R*_0_: the drop radius at the apex of the pendant drop) when the drop size is of the same order of magnitude as the needle diameter, and that the measured value approach the correct value as *B* approaches one^50^ (one could expect that the experimental precision will be optimized when the drop is most “swelled,” that is, when the droplet is on the verge of detaching off from the needle tip due to gravity, that is, when *B* = 1). We measured the value of tension as a function of *B* by using the software, OpenDrop, developed by Michael Neeson, Joe Berry and Rico Tabor. We extrapolated the data thus obtained to the value at *B* = 1 to have a pragmatic value, *γ* = 0.78 mN/m, because it was experimentally difficult to approach *B* = 1. This is possibly because the tension is significantly small, which might lead to an extra error in the measurement.

Even though the measurement of the interfacial tension contains an extra error and our analysis numerically depends on the measured value, this does not bring any uncertainties in the present arguments at the level of scaling laws. We explain this by an example. Introducing the experimentally measured value of surface tension *γ*_*m*_, we define a numerical coefficient *β* as *γ* = *β*^2^*γ*_*m*_ and the corresponding capillary length 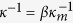. With these “measured” quantities, Eq. (8) can be expressed as 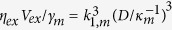 with 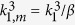. By noting that the values of the interfacial tension and capillary number used in Fig. 2(c) that experimentally confirms the relation Eq. (8) are in fact not *γ* and *κ*^−1^ but *γ*_*m*_ and 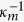, respectively, the coefficient we determined from Fig. 2(c) is in fact not *k*_1_ but *k*_1,*m*_. However, since Eq. (10) can be expressed as 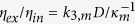 with *k*_3,*m*_ = *k*_3_/*β*, the phase boundary line 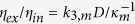 on the 
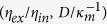
 space and the line *η*_*ex*_/*η*_*in*_ = *k*_3_*D*/*κ*^−1^ on the (*η*_*ex*_/*η*_*in*_, *D*/*κ*^−1^) space have the same physical meaning. From these reasons, a special care is needed when one compares the numerical coefficient obtained experimentally in the present study with more sophisticated experiments or calculations.

## Additional Information

**How to cite this article**: Yahashi, M. *et al*. Scaling crossover in thin-film drag dynamics of fluid drops in the Hele-Shaw cell. *Sci. Rep.* **6**, 31395; doi: 10.1038/srep31395 (2016).
